# Supplementary material for: Effects of different blood flow rates on filter and circuit life in non-anticoagulation CRRT: Protocol for a three-arm, single-blind randomized controlled trial (the Flow-CRRT Study)
Source: PLoS One. 2025 Aug 22;20(8):e0330845. doi: 10.1371/journal.pone.0330845 (PMC12373228; doi:10.1371/journal.pone.0330845)
Supplement: S2 File — (DOC) [file pone.0330845.s002.doc]

内 部 资 料

注 意 保 密

**生物医学伦理研究方案**

**（干预性临床研究）**

**不同体外循环血流速度对无抗凝CRRT滤器寿命影响的随机对照试验**

研究单位：四川大学华西医院

项目负责人（签名）：赵宇亮

承担科室：肾内科

联系电话：13880801130

组长单位： 无

参加单位： 无

研究年限：

2024年6月 —— 2025年5月

版本号：V3.0

版本日期：2024年6月3日

方 案 摘 要

| **研究设计**  **(可多选)** | **□病例对照研究 □队列研究 □横断面研究**  **☑随机对照研究 ☑应用盲法 □其他：** |
| --- | --- |
| **研究类型**  **（请根据项目类型勾选）** | **（A类：高风险）**  □Ⅲ类临床新技术（安全性、有效性确切，技术难度大、风险高）  □ 特殊人群研究（儿童、孕妇、智力低下者、精神障碍受试者等）  □ 超药物说明书研究（□超适应症 □超给药途径 □超剂量 □超年龄  □超禁忌症 □超人群 □其他，请说明： ）  □ 超器械说明书研究（□超适应症 □使用范围 □超禁忌症 □超人群  □其他，请说明： ）  □ 其他（研究者判定，请说明： ）  **（B类：中风险）**  □ 上市后生物制剂研究（预防用和治疗用）  □ 上市后治疗性疫苗研究  □ 上市后罕见病药物研究  □ Ⅱ类临床新技术（安全性、有效性确切，有一定技术难度，有一定医疗风险和伦理风险）  □ 其他（研究者判定，请说明： ）  **（C类：低风险）**  □ 已上市5年药物研究（包括化药、仿制药等）  □ 已上市器械研究（含AI，影像软件）  ☑ Ⅰ类临床新技术（安全性、有效性确切，技术难度低、几乎不存在伦理风险的医疗技术）  □ 其他（研究者判定，请说明： ） |
| **病例总数** | 486 |
| **风险/受益分析** | 血流量调整影响滤器寿命，不会对患者带来风险 |
| **风险判断** | □不大于最小风险 ☑大于最小风险  最小风险：指试验中预期风险的可能性和程度不大于日常生活、或进行常规体格检查或心理测试的风险 |

一、研究背景

连续性肾脏替代治疗（CRRT）作为一种治疗急性肾损伤（AKI）的有效手段，因其连续性、温和性和对血流动力学影响小等优点，被广泛应用于临床治疗中。CRRT的疗效不仅与治疗模式有关，还与滤器的使用寿命紧密相关。滤器的使用寿命越长，意味着更少的更换次数和更低的并发症风险，从而提高了治疗的安全性和经济性。

在CRRT中，抗凝是预防滤器凝血和延长滤器寿命的常规做法。然而，抗凝剂的使用存在出血风险，尤其是在有出血倾向的患者中。因此，无抗凝策略的应用逐渐受到重视，它旨在减少抗凝剂相关并发症，同时保持滤器的功能和寿命。

血流量是CRRT中一个关键的操作参数，它影响着滤器的凝血风险和治疗效果。较高的血流量可以减少血液在滤器中的滞留时间，从而降低滤器凝血的风险，减少血栓的形成；但对于血流动力学不稳定的患者，较高的血流速度也可能会导致血压下降、心输出量减少。适当的血流速度可以确保有效的滤过和清除代谢废物，从而有效改善肾功能。因此，研究血流量对无抗凝CRRT滤器寿命的影响，对于优化治疗方案、减少医疗资源消耗以及改善患者预后具有重要意义。

本研究旨在探讨不同血流量设置对无抗凝CRRT中滤器寿命的影响，以期为临床提供更为精确的治疗参数，改善AKI患者的治疗效果。

二、研究目的

1. 主要目的：

评估不同体外循环血流速度在CRRT中对无抗凝滤器寿命的影响。通过比较不同血流速度下CRRT滤器的寿命，确定最佳的体外循环血流速度，以最大程度地延长滤器的使用寿命，减少CRRT过程中的滤器更换次数，降低治疗成本，并改善患者的治疗效果。

2. 次要目的：

1）探讨不同血流速度下CRRT滤器的滤器性能和效果，包括肌酐清除率、尿素氮清除率和超滤速率等；

2）分析不同血流速度下患者的血流动力学参数和生理指标的变化，包括动脉血压等，以评估对患者生理状态的影响。

3）研究不同血流速度对CRRT过程中血栓形成、出血并发症等不良事件的影响，以评估安全性和可行性。

4）评估不同血流速度下CRRT治疗效果的临床结局，包括肾功能恢复情况、生存率和住院时间等，以确定最佳的治疗策略。

三、研究设计、方法与研究步骤

1. 研究设计

研究类型：单中心、单盲、前瞻性随机对照研究（RCT）

随机化分组方法：每位符合入选标准的患者在签署知情同意书并完成所有基线评估后，将由研究协调员使用计算机生成的随机数表被随机分配到三个组之一：低血流速度组、中等血流速度组或高血流速度组，随机分组将以1:1:1的比例进行，确保每组患者数量相等，从而最大限度地平衡三组之间的患者特征和潜在的混杂因素。这位研究协调员不参与患者的治疗和数据收集及分析工作，分组信息将被严格保密，以确保分组的公正性。每位患者的随机分组信息将记录在专用的研究数据库中，并有适当的安全措施保护数据不被未授权访问。此外，所有相关的研究人员将接受有关如何正确处理随机化信息的培训。在选择和治疗患者的过程中，只有授权的研究人员能够访问分组信息。这些授权人员包括主要研究者和分配治疗的医师，他们将通过加密的电子系统获取分组信息，以确保数据的安全和隐私。未授权的研究人员将无法获取分组信息，以维持单盲设计的完整性。所有与治疗和患者管理相关的决定将根据分组信息和临床情况由授权人员做出。

研究中心：四川大学华西医院

样本量计算：该研究的主要结局指标是滤器寿命，因此选用滤器消耗数量作为样本量。根据本中心回顾性+前瞻性队列研究数据，200mL/min血流速度组和250mL/min血流速度组滤器寿命差异最小，因此能通过这两组寿命的差距计算出可能的最大样本量。前期研究结果显示，200mL/min血流速度组的平均滤器寿命为28.09±21.15小时，250mL/min血流速度组的平均滤器寿命为35.51±17.97小时，选定显著性水平α为0.05、功效为0.90，我们应用软件PASS （Power Analysis and Sample Size Software）15计算出每组样本量N=153例，考虑失访以及拒访的情况5%计算，最终至少需要的各组滤器为162套，总计至少纳入486套滤器。

1. 研究方法

选取于四川大学华西医院符合纳入排除标准的CRRT的患者，将患者随机分为低血流速度组、中等血流速度组或高血流速度组。国际各大型单位对于无抗凝血流量的标准速度为250mL/min，本中心无抗凝血流量标准速度为200mL/min，对于血流动力学不稳定或由于管路压力过高的患者，本中心根据经验下调血流速度为150mL/min，这与既往研究以150mL/min定义低血流速度组是一致的[1]。综上所述，我们将150mL/min、200mL/min和250mL/min分别定义为低血流速度组、中等血流速度组和高血流速度组。

低血流速度组：调整CRRT血流量为150mL/min;

中等血流速度组：调整CRRT血流量为200mL/min;

高血流速度组：调整CRRT血流量为250mL/min;

余CRRT处方的制定及参数的调整根据患者实际情况、经验丰富的临床医师及CRRT专科医师决定。

若在CRRT过程中因血流量过高引起血管通路压力偏高需下调血流量，遵循以下原则：

逐步下调血流量： 以每次减少50mL/min的方式逐档下调，观察患者反应和滤器性能，直到达到稳定状态。评估和记录：在每次调整后，详细记录调整前后的血流量、滤器性能、患者的临床表现及原因。临床判断：根据患者的实际情况和临床判断，决定是否需要进一步调整，并确保患者的安全和治疗效果。后期在进行数据分析时，我们将分别使用意向性分析和按完成方案分析。

记录三组滤器寿命，凝血事件及低血压等其他CRRT相关负性事件的发生。

3. 研究步骤


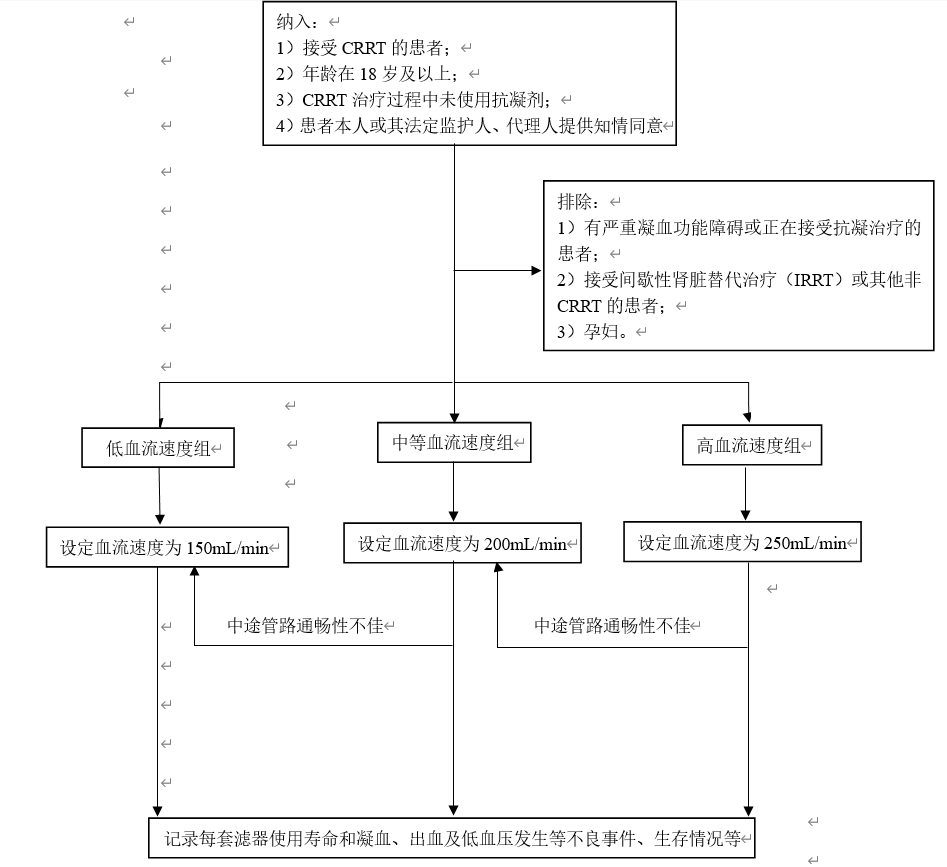


四、病例选择

1. 入选标准

1）接受CRRT的患者；

2）年龄在18岁及以上；

3）CRRT治疗过程中未使用抗凝剂；

4）患者本人或其法定监护人、代理人提供知情同意

2. 排除标准

1）有严重凝血功能障碍或正在接受抗凝治疗的患者；

2）接受间歇性肾脏替代治疗（IRRT）或其他非CRRT的患者；

3）孕妇。

3. 终止研究标准

1) 安全性问题

严重不良事件：如果在研究过程中观察到某一组与治疗相关的低血压、恶性心律失常、死亡等严重不良事件的发生率明显高于其他两组，或超出了预先设定的安全阈值应考虑中止该组研究。安全阈值的设定依据是根据既往类似研究的数据和临床专家的意见，以确保患者的安全。如出血事件的发生率高于10%，且比其他组高出5%或更多；血栓事件的发生率高于5%，且比另外两组高出3%或更多；感染事件的发生率超过15%，且比其他组高出5%或更多；心血管事件的发生率超过8%，且比另外两组高出4%或更多[2,3]。

2） 效果明显不足

中期分析：中期分析将在研究完成50%的患者入组后进行，由一名独立的统计学家进行，其对治疗分配不知情。

中期分析目标：评估不同血流速度组的滤器寿命、比较各组间的滤器堵塞率、评估不良事件的发生率。

中期分析指标：主要结局指标：滤器寿命（小时）；次要结局指标：滤器凝血率、不良事件发生率（出血、血栓形成等）。

中期分析方法：使用Kaplan-Meier生存分析法评估滤器寿命，并使用Log-rank检验比较不同组间的滤器寿命差异。采用卡方检验比较各组间滤器堵塞率和不良事件发生率的差异。

若中期分析结果显示：

任意一组的滤器寿命显著低于其他组（*P* < 0.05），并且不良事件发生率显著增加，则终止该组患者的入组；所有组间滤器寿命无显著差异（*P* > 0.05），且不良事件发生率无显著差异，则继续进行研究，直至完成所有患者的入组。

统计学家将向独立的数据和安全监测委员会（DSMB）报告，DSMB负责中期分析数据的审查和评估，并根据分析结果向研究者提出是否继续、修改或终止研究的建议[4][5]。

3） 统计显著性达成

提前达到研究目标：若中期分析结果显示滤器寿命在不同血流速度组之间的差异达到显著性水平（*P*<0.05），并且这种差异足以证明主要研究假设，可能提前结束研究。

4）受试者要求退出研究。

终止研究程序：研究负责人在接到DSMB建议后，应立即暂停新患者的入组，并通知所有研究中心；通知伦理委员会（IRB）和相关监管机构，提供终止原因和中期分析结果；为已入组患者提供适当的后续治疗和随访。

1. 可供选择的其他诊疗方法

1）设定更低或更高的血流量，如130mL/min或300mL/min；

2）采用肝素、局部枸橼酸等其他抗凝方式。

六、检测项目与检测时点

1）基线资料

患者入院时，详细记录患者的人口学特征、实验室指标、检查结果、原发病、合并症、合并用药、血压等；

2）滤器寿命：每次更换滤器时，记录每个滤器的使用时间，其中滤器将根据以下标准更换：滤器、导管或者静脉壶出现明显的凝血；滤器使用时间超过72小时（即使无其他问题）；患者因外出检查或者行其他治疗需要停机；达到治疗效果或者中止CRRT；

3）CRRT相关参数：

CRRT处方制定后，详细记录CRRT处方，包括超滤率、通路、模式等；若中途因管路性能不佳下调血流量或因凝血功能等原因调整抗凝方式，详细记录时间、调整量及调整前后变化等；

4）滤器性能和效果：

CRRT结束后，记录CRRT滤器清除性能，包括肌酐清除率、尿素氮清除率、胱抑素C和超滤速率等；

5）患者生理参数的变化：

在CRRT过程中，每2小时监测患者动脉血压，记录低血压等血流动力学不稳定事件的发生；

6）不良事件：

在CRRT过程中，随时观察并记录不良事件的发生情况，包括血栓形成、出血并发症等。

7）临床结局：

CRRT下机后，监测患者肾功能恢复情况，如肌酐和肾小球滤过率等的变化；出院后记录患者生存情况、住院时间等。

七、疗效评定标准

1）有效性评价：测量血流量对无抗凝CRRT滤器寿命的影响，记录每个患者的血流量、滤器使用时间以及可能的滤器故障或阻塞情况。

2）安全性评价：监测与血流量调整相关的不良事件，如血栓形成、出血或滤器故障，持续监测并记录每个患者发生的不良事件及其严重程度。使用统计方法分析不良事件的发生率，并与不同血流量水平进行比较，以评估其相关性。

八、不良事件的观察、记录和处置

在本研究中，不良事件指在CRRT过程中导致CRRT紧急下机或使患者出现生命危险，并且停止CRRT后情况有明显改善的事件。因此对CRRT不良事件的观察止于患者完全结束多疗程CRRT。

预计不良事件有：低血压、电解质失衡、出血或感染。

对不良事件与研究干预之间的关系进行判断通常基于：1）时间关联性：事件发生在CRRT上机后的特定时间内，或者排除其他原因导致的不良事件；2）已知的药物或器械副作用；3）事件是否在CRRT下机后消失或减轻。

不良事件的记录、处理与报告：1）记录：所有不良事件，无论大小或预期与否，均应详细记录在研究档案中。记录应包括事件的描述、严重性、开始和结束日期、处理措施、结果以及与研究治疗的关联性评估；2）处理：对于任何严重或意外的不良事件，立即采取适当的治疗措施，如发生低血压，进行减少超滤、补液升压等治疗，如发生过敏，及时予以地塞米松、抗组胺药物等，可能包括中止研究干预；3）报告：所有严重不良事件将在规定事件内报告给上级医师监管机构及伦理委员会。

严重不良事件：1）报告方法：研究人员应通过预定的报告表格将严重不良事件报告给研究赞助者和伦理审查委员会，同时按照当地法规和协议要求，上报给相关监管机构；2）处理措施：立即采取适当的医疗措施，必要时停止患者的研究参与，并进行详尽的诊断调查以确定事件的原因；3）随访方式：进行持续的监测和治疗直至事件解决或稳定。随访信息应包括后续治疗、医疗结果以及最终评估；4）时间：记录详细的时间线，包括事件发生、识别、报告和解决的日期。

九、研究的质量控制与质量保证

1.临床指标检测

所有临床指标的检测将在华西医院进行，确保操作一致性和结果的可比性。参与人员将接受专业培训，并按照标准操作程序（SOP）执行检测过程，以最大程度减少误差。

2.执行相关SOP

为确保研究的一致性，所有研究相关操作将按照事先制定的标准操作程序（SOP）执行。研究人员将在研究开始前接受培训，包括但不限于技术操作、设备使用、数据收集规范等方面，以确保操作的一致性和标准化。

3．研究者培训

所有研究人员都将接受系统的培训，包括研究目的、操作流程、伦理标准等方面的培训。培训将定期进行更新，以确保研究者对研究的了解程度和执行力保持在高水平。

4．受试者依从性

为了确保受试者的依从性，将建立定期的沟通和回访机制。研究人员将与受试者建立良好的沟通渠道，详细解释研究流程、重要性，鼓励并监测受试者的依从性。

5．数据收集整理分析

数据收集将采用统一的数据表格，确保数据的完整性和准确性。数据整理将由专业数据管理员进行，检查数据的完备性和逻辑性。数据分析将由经验丰富的统计分析人员完成，采用先进的分析方法确保结果的可信性。

6．研究监查

研究将接受独立的监查机制，由专业监查人员对研究的执行过程、数据的真实性和合规性进行定期监查。监查报告将及时反馈给研究团队，确保及时纠正任何潜在问题。

这些质量控制与监督措施的实施将有助于提高研究的可靠性和结果的科学性，确保研究的顺利进行。

十、数据安全监查

临床研究将根据风险大小制定相应的数据安全监察计划。所有不良事件均详细记录，恰当处理并追踪直到妥善解决或病情稳定，按照规定及时向伦理审查委员会、主管部门、申办者和药品监督管理部门报告严重不良事件与非预期事件等；主要研究者定期对所有不良事件进行累积性回顾，必要时召开研究者会议评估研究的风险与受益；双盲试验必要时可以进行紧急揭盲，以确保受试者安全与权益。

十一、统计学处理

采用SPSS软件（27.0, SPSS Inc. 美国）和R 4.2 (R Foundation for Statistical Computing, Vienna, Austria) 软件进行数据分析。

1）描述性统计分析：

对研究样本的基本特征进行描述，如人口学特征、实验室指标、基础疾病等；对不同血流速度组的滤器寿命、滤器性能指标、患者生理参数变化等进行描述性统计分析，包括平均值、标准差、中位数等。

2）生存分析：

使用生存分析方法（如Kaplan-Meier曲线）比较不同血流速度组之间滤器寿命的差异。

采用Cox比例风险模型等方法分析可能影响滤器寿命的因素，如血流速度、患者年龄、基础疾病等。

3）方差分析：

对滤器性能指标在不同血流速度组之间的差异进行方差分析。

4）安全性和不良事件分析：

使用卡方检验或Fisher确切检验比较不同血流速度组之间不良事件（如血栓形成、出血并发症）的发生率，以评估不同血流速度对CRRT治疗的安全性。

5）临床结局分析：

使用适当的统计方法比较不同血流速度组之间的临床结局，如肾功能恢复情况、生存率、住院时间等。

P值<0.05将被认为所检验的差别有统计意义。

十二、临床研究伦理原则与要求

临床研究将遵循世界医学大会《赫尔辛基宣言》和中华人民共和国国家卫生和计划生育委员会《涉及人的生物医学研究伦理审查办法》等相关规定，具体落实知情同意，保护隐私，研究免费与补偿，控制风险，特殊受试者保护和研究相关损害的赔偿原则与要求。在研究开始之前，由伦理审查委员会批准该试验方案后才实施临床研究。每一位受试者入选本研究前，研究者有责任向受试者或/和其法定代理人完整、全面地介绍本研究的目的、程序和可能的风险，并签署书面知情同意书，应让受试者知道他们参加临床研究完全是自愿的，他们可以拒绝参加或在试验的任何阶段随时退出本研究而不会受到歧视和报复，其医疗待遇与权益不受影响。知情同意书应作为临床研究文件保留备查，切实保护受试者的个人隐私与数据机密性。

十三、研究进度

2024年5月-2024年7月：伦理撰写及研究注册

2024年8月-2025年2月：研究实施

2025年3月-2025年5月：数据收集、分析与文章撰写

十四、参加人员

| **姓名** | **职称** | **专业** | **任务** | **GCP培训证书** |
| --- | --- | --- | --- | --- |
| 赵宇亮 | 副主任医师 | 肾脏病学 | 统筹安排 | 有 |
| 刘彩虹 | 研究生 | 肾脏病学 | 研究设计 | 无 |
| 黄永秀 | 研究生 | 肾脏病学 | 数据收集 | 无 |
| 韦伟 | 研究生 | 肾脏病学 | 数据分析 | 无 |
| 宋国姣 | 本科生 | 临床医学 | 数据分析 | 无 |
| 王芳 | 主管护师 | 护理学 | 设备操作 | 有 |
| 张凌 | 主任医师 | 肾脏病学 | 指导设计 | 有 |

1. 主要参考文献

1. Fealy N, Aitken L, du Toit E, Lo S, Baldwin I. Faster Blood Flow Rate Does Not Improve Circuit Life in Continuous Renal Replacement Therapy: A Randomized Controlled Trial. Crit Care Med. 2017;45:e1018–25.

2. Connolly SJ, Ezekowitz MD, Yusuf S, Eikelboom J, Oldgren J, Parekh A, et al. Dabigatran versus warfarin in patients with atrial fibrillation. N Engl J Med. 2009;361:1139–51.

3. Heart Outcomes Prevention Evaluation Study Investigators, Yusuf S, Sleight P, Pogue J, Bosch J, Davies R, et al. Effects of an angiotensin-converting-enzyme inhibitor, ramipril, on cardiovascular events in high-risk patients. N Engl J Med. 2000;342:145–53.

4. Ciolino JD, Kaizer AM, Bonner LB. Guidance on interim analysis methods in clinical trials. Journal of Clinical and Translational Science. 2023;7:e124.

5. Interim analysis – GUIDANCE FOR CLINICAL TRIAL PROTOCOLS [Internet]. [cited 2024 Jun 1]. Available from: https://spirit-statement.org/interim-analysis/
