# Supplementary material for: Effects of different blood flow rates on filter and circuit life in non-anticoagulation CRRT: Protocol for a three-arm, single-blind randomized controlled trial (the Flow-CRRT Study)
Source: PLoS One. 2025 Aug 22;20(8):e0330845. doi: 10.1371/journal.pone.0330845 (PMC12373228; doi:10.1371/journal.pone.0330845)
Supplement: S3 File — (DOCX) [file pone.0330845.s003.docx]

**Biomedical Ethics Research Protocol**

**(Interventional Clinical Study)**

**Impact of Different Extracorporeal Blood Flow Rates on Filter Lifespan in Anticoagulant-Free CRRT: A Randomized Controlled Trial**

**Research Unit:** West China Hospital, Sichuan University
**Principal Investigator (Signature):** Zhao Yuliang
**Department:** Nephrology
**Contact Number:** +86-13880801130
**Lead Unit:** None
**Participating Units:** None
**Study Period:** June 2024 — May 2025
**Version Number:** V3.0
**Version Date:** June 3, 2024

**Abstract of the Protocol**

|Study Design|□ Case-Control Study □ Cohort Study □ Cross-Sectional Study|
**☑**Randomized Controlled Trial (Blinding Applied) □ Other:|
|Study Type| (Category A: High Risk)|
|(Please select according to the project type)|□ Class III Clinical New Technology (Definite safety and efficacy, high technical difficulty and risk)|
|□ Special Population Study (Children, pregnant women, individuals with intellectual disabilities,

patients with mental disorders, etc.)|
|□ Off-Label Drug Study (□ Off-indication □ Off-route of administration □ Off-dose □ Off-age|
|□ Off-contraindication □ Off-population □ Other, please specify: )|
|□ Off-Label Device Study (□ Off-indication □ Off-use range □ Off-contraindication □ Off-population)|
|□ Other (Please specify: )|
|□ (Category B: Medium Risk)|
|□ Post-marketing Biologics Study (Preventive and therapeutic)|
|□ Post-marketing Therapeutic Vaccine Study|
|□ Post-marketing Orphan Drug Study|
|□ Class II Clinical New Technology (Definite safety and efficacy, certain technical difficulty, certain medical and ethical risks)|
|□ Other (Please specify: )|
|□ (Category C: Low Risk)|
|□ Post-marketing Drug Study (Including chemical drugs, generic drugs, etc.)|
|□ Post-marketing Device Study (Including AI, imaging software)|
|**☑**(Class I Clinical New Technology (Definite safety and efficacy, low technical difficulty, virtually no ethical risk in medical technology)|
|□ Other (Please specify: )|
|Total Number of Cases|486|
|Risk/Benefit Analysis|Adjusting blood flow rate affects filter lifespan without posing risks to patients|
|Risk Assessment|□ No greater than minimal risk (**☑**Greater than minimal risk)|
|Minimal risk: Refers to the possibility and extent of risks in the trial not exceeding those of daily life, or undergoing routine physical examination or psychological testing|

**I. Research Background**

Continuous renal replacement therapy (CRRT) is an effective treatment for acute kidney injury (AKI). Due to its continuous nature, mildness, and minimal impact on hemodynamics, it has been widely applied in clinical practice. The efficacy of CRRT is not only related to the treatment modality but also closely associated with the lifespan of the filter. A longer filter lifespan means fewer replacements and a lower risk of complications, thereby enhancing the safety and cost-effectiveness of the treatment.

Anticoagulation is a conventional practice in CRRT to prevent filter clotting and prolong filter lifespan. However, the use of anticoagulants carries a risk of bleeding, especially in patients with a tendency to bleed. Therefore, anticoagulant-free strategies have gained increasing attention, aiming to reduce anticoagulant-related complications while maintaining filter function and lifespan.

Blood flow rate is a critical operational parameter in CRRT, influencing both filter clotting risk and treatment efficacy. A higher blood flow rate can reduce the dwell time of blood in the filter, thereby lowering the risk of filter clotting and thrombus formation. However, for hemodynamically unstable patients, a higher flow rate may lead to decreased blood pressure and reduced cardiac output. An appropriate blood flow rate can ensure effective filtration and clearance of metabolic waste, thereby improving renal function. Thus, studying the impact of blood flow rate on filter lifespan in anticoagulant-free CRRT is of significant importance for optimizing treatment protocols, reducing medical resource consumption, and improving patient outcomes.

This study aims to investigate the impact of different blood flow rate settings on filter lifespan in anticoagulant-free CRRT, in order to provide more precise treatment parameters for clinical practice and improve the therapeutic outcomes for AKI patients.

**II. Research Objectives**

1. **Primary Objective:** To evaluate the impact of different extracorporeal blood flow rates on filter lifespan in CRRT without anticoagulation. By comparing filter lifespans under different blood flow rates, the optimal extracorporeal blood flow rate will be determined to maximize filter lifespan, reduce the number of filter replacements during CRRT, lower treatment costs, and improve patient outcomes.
2. **Secondary Objectives:**
   1. To explore the performance and efficacy of CRRT filters under different blood flow rates, including creatinine clearance, urea nitrogen clearance, and ultrafiltration rate.
   2. To analyze changes in hemodynamic parameters and physiological indices of patients under different blood flow rates, including arterial blood pressure, to assess the impact on patient physiological status.
   3. To study the impact of different blood flow rates on adverse events during CRRT, such as thrombus formation and bleeding complications, to evaluate safety and feasibility.
   4. To assess the clinical outcomes of CRRT treatment under different blood flow rates, including renal function recovery, survival rate, and hospital stay duration, to determine the optimal treatment strategy.

**III. Research Design, Methods, and Steps**

1. **Research Design**
   - **Study Type:** Single-center, single-blind, prospective randomized controlled trial (RCT)
   - **Randomization Method:** Each patient meeting the inclusion criteria will be randomly assigned to one of three groups (low blood flow rate group, medium blood flow rate group, or high blood flow rate group) by a study coordinator using a computer-generated random number table. Randomization will be conducted in a 1:1:1 ratio to ensure equal numbers of patients in each group, thereby maximizing the balance of patient characteristics and potential confounding factors across the groups. The study coordinator will not be involved in patient treatment or data collection and analysis. Group assignment information will be strictly confidential to ensure fairness. Each patient's randomization information will be recorded in a dedicated study database with appropriate security measures to prevent unauthorized access. All relevant researchers will receive training on the proper handling of randomization information. During patient selection and treatment, only authorized researchers will have access to group assignment information. These authorized personnel, including the principal investigator and the physician assigning treatment, will access the information through an encrypted electronic system to ensure data security and privacy. Unauthorized researchers will not have access to group assignment information to maintain the integrity of the single-blind design. All treatment and patient management decisions will be made by authorized personnel based on group assignment information and clinical conditions.
   - **Research Center:** West China Hospital, Sichuan University
   - **Sample Size Calculation:** The primary outcome measure of this study is filter lifespan. Therefore, filter consumption quantity is selected for sample size calculation. Based on retrospective and prospective cohort study data from our center, the smallest difference in filter lifespan was observed between the 200 mL/min and 250 mL/min blood flow rate groups. Thus, the maximum possible sample size can be calculated based on the lifespan difference between these two groups. Previous study results showed that the average filter lifespan was 28.09 ± 21.15 hours in the 200 mL/min blood flow rate group and 35.51 ± 17.97 hours in the 250 mL/min blood flow rate group. With a significance level (α) set at 0.05 and power at 0.90, the software PASS (Power Analysis and Sample Size Software) version 15 was used to calculate a sample size of N = 153 per group. Considering a dropout and refusal rate of 5%, the final number of filters required per group is at least 162, totaling at least 486 filters.
2. **Research Methods**

Patients undergoing CRRT at West China Hospital who meet the inclusion and exclusion criteria will be randomly divided into three groups: low blood flow rate group, medium blood flow rate group, and high blood flow rate group. The international standard blood flow rate for anticoagulant-free CRRT is 250 mL/min, while the standard blood flow rate for anticoagulant-free CRRT at our center is 200 mL/min. For hemodynamically unstable patients or those with high circuit pressure, our center reduces the blood flow rate to 150 mL/min based on experience, which is consistent with previous studies defining 150 mL/min as the low blood flow rate group [1]. Therefore, we define 150 mL/min, 200 mL/min, and 250 mL/min as the low, medium, and high blood flow rate groups, respectively.

- **Low Blood Flow Rate Group:** Adjust the CRRT blood flow rate to 150 mL/min.
- **Medium Blood Flow Rate Group:** Adjust the CRRT blood flow rate to 200 mL/min.
- **High Blood Flow Rate Group:** Adjust the CRRT blood flow rate to 250 mL/min.

The remaining CRRT prescription and parameter adjustments will be determined based on the patient's actual condition, experienced clinical physicians, and CRRT specialists. If during CRRT, high blood flow rate causes high vascular access pressure and requires a reduction in blood flow rate, the following principles will be followed:

- **Stepwise Reduction of Blood Flow Rate:** The blood flow rate will be reduced in increments of 50 mL/min, observing the patient's response and filter performance until a stable state is achieved.
- **Assessment and Recording:** After each adjustment, detailed records will be kept of the blood flow rate before and after adjustment, filter performance, patient's clinical manifestations, and reasons. Clinical judgment will be made based on the patient's actual condition to decide whether further adjustment is needed and to ensure patient safety and treatment efficacy. During data analysis, intention-to-treat analysis and per-protocol analysis will be used separately.
- **Record Filter Lifespan and Adverse Events:** Record the lifespan of the filters in the three groups, as well as the occurrence of clotting events and other negative events related to CRRT such as hypotension.

1. **Research Steps**

[Diagram]

**IV. Case Selection**

1. **Inclusion Criteria**
   1. Patients undergoing CRRT;
   2. Age 18 years or older;
   3. No anticoagulant used during CRRT;
   4. Informed consent provided by the patient or their legal guardian/agent.
2. **Exclusion Criteria**
   1. Patients with severe coagulation disorders or undergoing anticoagulant therapy;
   2. Patients receiving intermittent renal replacement therapy (IRRT) or other non-CRRT treatments;
   3. Pregnant women.
3. **Criteria for Study Termination**
   1. **Safety Issues:** Serious adverse events: If during the study, the incidence of serious adverse events related to treatment such as hypotension, malignant arrhythmias, or death is significantly higher in one group compared to the other two groups, or exceeds the predefined safety threshold, consideration should be given to terminating that group's study. The safety threshold is set based on data from similar previous studies and the opinions of clinical experts to ensure patient safety. For example, if the incidence of bleeding events exceeds 10% and is 5% or more higher than the other groups; the incidence of thrombotic events exceeds 5% and is 3% or more higher than the other two groups; the incidence of infectious events exceeds 15% and is 5% or more higher than the other groups; the incidence of cardiovascular events exceeds 8% and is 4% or more higher than the other two groups [2,3].
   2. **Inadequate Effectiveness:** Interim analysis: Interim analysis will be conducted after 50% of the patients have been enrolled, performed by an independent statistician who is blinded to treatment allocation.
   3. **Interim Analysis Objectives:** To assess filter lifespan in different blood flow rate groups, compare filter blockage rates among groups, and evaluate the incidence of adverse events.
   4. **Interim Analysis Indicators:** Primary outcome measure: Filter lifespan (hours); Secondary outcome measures: Filter clotting rate, incidence of adverse events (bleeding, thrombus formation, etc.).
   5. **Interim Analysis Method:** Kaplan-Meier survival analysis will be used to assess filter lifespan, and the Log-rank test will be used to compare differences in filter lifespan among different groups. The chi-square test will be used to compare differences in filter blockage rates and adverse event incidence among groups.
   6. If the interim analysis results show that the filter lifespan in any group is significantly lower than the other groups (P < 0.05), and the incidence of adverse events is significantly increased, enrollment of patients in that group will be terminated. If there is no significant difference in filter lifespan among all groups (P > 0.05), and no significant difference in adverse event incidence, the study will continue until all patients have been enrolled.
   7. The statistician will report to the independent Data and Safety Monitoring Board (DSMB), which will review and assess the interim analysis data and provide recommendations to the researchers on whether to continue, modify, or terminate the study [4][5].
   8. **Achievement of Statistical Significance:** If the interim analysis shows a significant difference in filter lifespan among different blood flow rate groups (P < 0.05), and this difference is sufficient to prove the primary research hypothesis, the study may be terminated early.
   9. **Participant Request to Withdraw:** If a participant requests to withdraw from the study.

**Termination Procedure:** Upon receiving the DSMB's recommendation, the study leader should immediately suspend enrollment of new patients and notify all research centers; inform the Institutional Review Board (IRB) and relevant regulatory authorities, providing the reasons for termination and the interim analysis results; provide appropriate follow-up treatment and follow-up for patients already enrolled.

**Alternative Diagnostic and Treatment Options:**

1. Setting a lower or higher blood flow rate, such as 130 mL/min or 300 mL/min;
2. Using other anticoagulation methods such as heparin or regional citrate anticoagulation.

**VI. Detection Items and Time Points**

1. **Baseline Data:** At the time of patient admission, detailed records will be kept of demographic characteristics, laboratory indicators, examination results, primary disease, comorbidities, concomitant medications, blood pressure, etc.
2. **Filter Lifespan:** Each time a filter is replaced, the usage time of each filter will be recorded. Filters will be replaced based on the following criteria: obvious clotting in the filter, catheter, or venous chamber; filter usage time exceeding 72 hours (even if there are no other issues); patient needs to stop the machine for an outside examination or other treatments; treatment goals are achieved or CRRT is terminated.
3. **CRRT-Related Parameters:** After the CRRT prescription is established, detailed records will be kept of the CRRT prescription, including ultrafiltration rate, access, mode, etc. If the blood flow rate is reduced due to poor circuit performance or the anticoagulation method is adjusted due to coagulation function, the time, adjustment amount, and changes before and after adjustment will be recorded in detail.
4. **Filter Performance and Efficacy:** After CRRT is completed, the filter clearance performance will be recorded, including creatinine clearance, urea nitrogen clearance, cystatin C, and ultrafiltration rate.
5. **Patient Physiological Parameter Changes:** During CRRT, arterial blood pressure will be monitored every 2 hours, and the occurrence of hypotension and other hemodynamic instability events will be recorded.
6. **Adverse Events:** During CRRT, any adverse events that occur will be observed and recorded, including thrombus formation, bleeding complications, etc.
7. **Clinical Outcomes:** After CRRT is stopped, the patient's renal function recovery will be monitored, such as changes in creatinine and glomerular filtration rate. After discharge, the patient's survival status and hospital stay duration will be recorded.

**VII. Efficacy Evaluation Criteria**

1. **Efficacy Evaluation:** Measure the impact of blood flow rate on filter lifespan in anticoagulant-free CRRT, recording each patient's blood flow rate, filter usage time, and any potential filter failures or blockages.
2. **Safety Evaluation:** Monitor adverse events related to blood flow rate adjustments, such as thrombus formation, bleeding, or filter failure, continuously monitoring and recording the occurrence and severity of adverse events in each patient. Use statistical methods to analyze the incidence of adverse events and compare them with different blood flow levels to assess their correlation.

**VIII. Observation, Recording, and Management of Adverse Events**

In this study, adverse events refer to events during CRRT that cause CRRT to be urgently stopped or pose a life-threatening risk to the patient, with significant improvement after CRRT is stopped. Therefore, the observation of CRRT adverse events ends when the patient completely finishes multiple courses of CRRT.

Anticipated adverse events include hypotension, electrolyte imbalance, bleeding, or infection. The relationship between adverse events and study interventions is typically judged based on: 1) Temporal association: The event occurs within a specific time after CRRT initiation, or other causes of adverse events are excluded; 2) Known side effects of drugs or devices; 3) Whether the event disappears or alleviates after CRRT is stopped.

**Recording, Management, and Reporting of Adverse Events:**

1. **Recording:** All adverse events, regardless of severity or expectation, should be meticulously documented in the study records. The documentation should include a description of the event, its severity, start and end dates, management measures, outcomes, and an assessment of the association with the study treatment.
2. **Management:** For any serious or unexpected adverse events, appropriate therapeutic measures should be immediately taken. For instance, in the case of hypotension, measures such as reducing ultrafiltration and administering fluids to raise blood pressure should be implemented. In the event of an allergic reaction, timely administration of dexamethasone and antihistamines may be necessary, which could also include discontinuing the study intervention.
3. **Reporting:** All serious adverse events will be reported to the supervising physician regulatory authorities and the ethics committee within the stipulated timeframe. Additionally, in accordance with local regulations and protocol requirements, these events will be reported to the relevant regulatory bodies.

**Serious Adverse Events:**

1. **Reporting Method:** Researchers should utilize a pre-established reporting form to notify the study sponsor and the ethics review committee of serious adverse events. Concurrently, in compliance with local regulations and protocol mandates, these events should be reported to the relevant regulatory authorities.
2. **Management Measures:** Immediate and appropriate medical actions should be taken. If necessary, the patient's participation in the study should be halted, and a thorough diagnostic investigation should be conducted to determine the cause of the event.
3. **Follow-up Method:** Continuous monitoring and treatment should be provided until the event is resolved or stabilized. Follow-up information should encompass subsequent treatments, medical outcomes, and a final assessment.
4. **Timing:** A detailed timeline should be maintained, documenting the dates of event occurrence, identification, reporting, and resolution.

**IX. Quality Control and Assurance of the Study**

1. **Clinical Indicator Testing:** All clinical indicator tests will be conducted at West China Hospital to ensure consistency in operations and comparability of results. Participants will receive professional training and will follow standard operating procedures (SOP) during the testing process to minimize errors as much as possible.
2. **Implementation of SOPs:** To ensure consistency in the study, all study-related operations will be carried out in accordance with the pre-established SOPs. Researchers will receive training before the start of the study, including but not limited to technical operations, equipment use, and data collection standards, to ensure consistency and standardization of operations.
3. **Researcher Training:** All researchers will receive systematic training, including the study objectives, operational procedures, and ethical standards. Training will be regularly updated to ensure that researchers maintain a high level of understanding and execution of the study.
4. **Participant Compliance:** To ensure participant compliance, a regular communication and follow-up mechanism will be established. Researchers will establish good communication channels with participants, thoroughly explain the study process and its importance, and encourage and monitor participant compliance.
5. **Data Collection, Organization, and Analysis:** Data collection will use a unified data form to ensure the completeness and accuracy of the data. Data organization will be carried out by professional data administrators to check the completeness and logical consistency of the data. Data analysis will be completed by experienced statistical analysts using advanced analytical methods to ensure the credibility of the results.
6. **Study Monitoring:** The study will be subject to an independent monitoring mechanism, with professional monitors regularly checking the execution of the study, the authenticity of the data, and compliance. Monitoring reports will be promptly fed back to the research team to ensure that any potential issues are corrected in a timely manner.

The implementation of these quality control and supervision measures will help enhance the reliability of the study and the scientific nature of the results, ensuring the smooth progress of the study.

**X. Data Safety Monitoring**

Clinical studies will develop a corresponding data safety monitoring plan based on the level of risk. All adverse events will be meticulously documented, appropriately managed, and followed up until they are satisfactorily resolved or stabilized. Serious adverse events and unexpected events will be reported in a timely manner to the ethics review committee, competent authorities, sponsors, and drug regulatory departments in accordance with regulations. The principal investigator will conduct periodic cumulative reviews of all adverse events and convene investigator meetings as necessary to assess the risks and benefits of the study. In the case of a double-blind trial, emergency unblinding may be necessary to ensure the safety and rights of the participants.

**XI. Statistical Analysis**

SPSS software (version 27.0, SPSS Inc., USA) and R version 4.2 (R Foundation for Statistical Computing, Vienna, Austria) will be used for data analysis.

1. **Descriptive Statistical Analysis:** Basic characteristics of the study sample will be described, including demographic features, laboratory indicators, underlying diseases, etc. Descriptive statistical analysis will also be conducted on filter lifespan, filter performance indicators, and changes in patient physiological parameters across different blood flow rate groups, including mean values, standard deviations, medians, etc.
2. **Survival Analysis:** Survival analysis methods (e.g., Kaplan-Meier curves) will be used to compare differences in filter lifespan among different blood flow rate groups. Cox proportional hazards models will be employed to analyze factors that may affect filter lifespan, such as blood flow rate, patient age, underlying diseases, etc.
3. **Analysis of Variance (ANOVA):** Differences in filter performance indicators among different blood flow rate groups will be analyzed using ANOVA.
4. **Safety and Adverse Event Analysis:** The incidence of adverse events (e.g., thrombus formation, bleeding complications) among different blood flow rate groups will be compared using chi-square tests or Fisher's exact tests to assess the safety of CRRT treatment at different blood flow rates.
5. **Clinical Outcome Analysis:** Appropriate statistical methods will be used to compare clinical outcomes, such as renal function recovery, survival rate, and hospital stay duration, among different blood flow rate groups.

A P-value of less than 0.05 will be considered statistically significant.

**XII. Ethical Principles and Requirements of Clinical Research**

Clinical research will adhere to the World Medical Association's Declaration of Helsinki and the relevant regulations of the National Health and Family Planning Commission of the People's Republic of China, "Ethical Review Measures for Biomedical Research Involving Human Beings," among others. Specific implementation will include informed consent, protection of privacy, free research participation and compensation, risk control, protection of special participants, and compensation for research-related damages. The trial protocol will only be implemented after approval by the ethics review committee. Before each participant is enrolled in the study, the researcher has the responsibility to fully and comprehensively introduce the purpose, procedures, and potential risks of the study to the participant or their legal representative, and obtain written informed consent. Participants should be informed that their participation in the clinical research is entirely voluntary, and they have the right to refuse to participate or withdraw from the study at any stage without facing discrimination or retaliation, and their medical treatment and rights will not be affected. The informed consent form should be retained as a clinical research document for future reference, and the personal privacy and data confidentiality of participants should be strictly protected.

**XIII. Study Timeline**

- May 2024 — July 2024: Ethical review and study registration
- August 2024 — February 2025: Study implementation
- March 2025 — May 2025: Data collection, analysis, and manuscript writing

**XIV. Participants**

| **Name** | **Title** | **Specialty** | **Tasks** | **GCP Training Certificate** |
| --- | --- | --- | --- | --- |
| Zhao Yuliang | Deputy Chief Physician | Nephrology | Overall coordination | Yes |
| Liu Caihong | Graduate Student | Nephrology | Study design | No |
| Huang Yongxiu | Graduate Student | Nephrology | Data collection | No |
| Wei Wei | Graduate Student | Nephrology | Data analysis | No |
| Song Guojiao | Undergraduate Student | Clinical Medicine | Data analysis | No |
| Wang Fang | Chief Nurse | Nursing | Equipment operation | Yes |
| Zhang Ling | Chief Physician | Nephrology | Guidance on study design | Yes |

**Main References**

1. Fealy N, Aitken L, du Toit E, Lo S, Baldwin I. Faster Blood Flow Rate Does Not Improve Circuit Life in Continuous Renal Replacement Therapy: A Randomized Controlled Trial. Crit Care Med. 2017;45:e1018–25.
2. Connolly SJ, Ezekowitz MD, Yusuf S, Eikelboom J, Oldgren J, Parekh A, et al. Dabigatran versus warfarin in patients with atrial fibrillation. N Engl J Med. 2009;361:1139–51.
3. Heart Outcomes Prevention Evaluation Study Investigators, Yusuf S, Sleight P, Pogue J, Bosch J, Davies R, et al. Effects of an angiotensin-converting-enzyme inhibitor, ramipril, on cardiovascular events in high-risk patients. N Engl J Med. 2000;342:145–53.
4. Ciolino JD, Kaizer AM, Bonner LB. Guidance on interim analysis methods in clinical trials. Journal of Clinical and Translational Science. 2023;7:e124.
5. Interim analysis – GUIDANCE FOR CLINICAL TRIAL PROTOCOLS [Internet]. [cited 2024 Jun 1]. Available from: <https://spirit-statement.org/interim-analysis/>
